# Supplementary material for: Candidate Genes for Freezing and Drought Tolerance Selected on the Basis of Proteome Analysis in Doubled Haploid Lines of Barley
Source: Int J Mol Sci. 2020 Mar 17;21(6):2062. doi: 10.3390/ijms21062062 (PMC7139788; doi:10.3390/ijms21062062)
Supplement: Supplementary file 1 [file ijms-21-02062-s001.pdf]

Table S1. Comparison of changes in transcripts and proteins accumulation after cold acclimation and drought treatment

Gene	Line	Direction of change in transcript accumulation after stress treatment	Direction of change in protein accumulation after stress treatment**	Reference for protein accumulation data*
14-3-3a	DH534	1.36-fold increase	not analyzed	[8]
	DH602	1.05-fold decrease	3-fold increase	[8]
	DH158	1.3-fold increase	no change	[8]
	DH575	2.88-fold increase	12-fold decrease	[8]
Elongation factor 1 alpha	DH534	1.09-fold increase	not analyzed	[8]
	DH602	1.84-fold increase	no change	[8]
	DH158	1.11-fold increase	no change	[8]
	DH575	1.67-fold increase	21-fold decrease	[8]
Ferredoxin-NADP reductase	DH534	1.78-fold decrease	not analyzed	[8]
	DH602	2.2-fold decrease	1.5-fold increase	[8]
	DH158	1.96-fold decrease	1.5-fold decrease	[8]
	DH575	2.06-fold increase	13-fold increase	[8]
β-fructofuranosidase	DH534	12.95-fold decrease	not analyzed	[8]
	DH602	37.17-fold decrease	13-fold increase	[8]
	DH158	10.47-fold decrease	1.5-fold decrease	[8]
	DH575	1.84-fold decrease	12-fold decrease	[8]
CBF4B	DH534	1.56-fold increase	not analyzed	[8]
	DH602	1.38-fold increase	13-fold increase	[8]
	DH158	1.21-fold increase	1.5-fold decrease	[8]
	DH575	2.87-fold increase	12-fold decrease	[8]
CBF2A	DH534	4.61-fold decrease	not analyzed	[8]
	DH602	3.48-fold	13-fold increase	[8]

		decrease		
	DH158	4.71-fold decrease	1.5-fold decrease	[8]
	DH575	2.27-fold decrease	12-fold decrease	[8]
Actin	DH534	2.21-fold decrease	no change	[9]
	DH561	1.53-fold decrease	0,05-fold decrease	[9]
	DH435	1.25-fold decrease	no change	[9]
	DH602	1.57-fold decrease	0.4-fold decrease	[9]
Triosephosphate isomerase	DH534	4.64-fold decrease	no change	[9]
	DH561	3.19-fold decrease	no change	[9]
	DH435	2.36-fold decrease	no change	[9]
	DH602	1.95-fold decrease	12.2-fold increase	[9]
Pfam14200	DH534	2.5-fold decrease	no change	[9]
	DH561	2.07-fold decrease	no change	[9]
	DH435	1.18-fold decrease	no change	[9]
	DH602	2.35-fold decrease	no change	[9]
GroEs	DH534	1.79-fold decrease	no change	[9]
	DH561	1.76-fold decrease	no change	[9]
	DH435	3.16-fold decrease	no change	[9]
	DH602	1.64-fold decrease	no change	[9]
periplasmic serine protease	DH534	3.95-fold decrease	no change	[9]
	DH561	2.04-fold decrease	no change	[9]
	DH435	1.67-fold decrease	0.5-fold decrease	[9]
	DH602	1.44-fold decrease	0.4-fold decrease	[9]
Transketolase	DH534	3.19-fold decrease	no change	[9]
	DH561	2.55-fold decrease	no change	[9]

	DH435	1.65-fold decrease	2.9-fold increase	[9]
	DH602	1.55-fold decrease	0.5-fold decrease	[9]

* The data of the direction of change in protein accumulation after stress treatment were used to present the tolerant vs susceptible lines ratio in those papers
** Where ‘no change’ is indicated, the difference between susceptible and tolerant lines were observed after stress treatment, but there were no changes before and after stress-treatment in a particular line
